# Comparison of coronal and sagittal pelvic-lower limb alignment after open wedge distal tibial tuberosity osteotomy and total knee arthroplasty using the EOS imaging system

**DOI:** 10.1016/j.asmart.2026.08.002

**Published:** 2026-08-19

**Authors:** Youngji Kim, Jun Tomura, Kenji Nakamura, Keiichi Yoshida, Shinnosuke Hada, Jun Shiozawa, Haruka Kaneko, Yoshitomo Saita, Mitsuaki Kubota, Muneaki Ishijima

**Affiliations:** a Department of Orthopedics, Juntendo University, Faculty of Medicine, Tokyo, Japan; b Department of Medicine for Orthopaedics and Motor Organ, Juntendo University, Graduate School of Medicine, Tokyo, Japan

**Keywords:** EOS system, Open wedge distal tuberosity tibial osteotomy, Pelvic-lower limb alignment, Sagittal alignment, Total knee arthroplasty

## Abstract

**Background:**

The evaluation in pelvic-lower limb alignment compared to both open wedge distal tuberosity osteotomy (OWDTO) and total knee arthroplasty (TKA) using the EOS imaging system remain unclear. The primary objective of this study is to evaluate the pelvic–lower limb alignment after OWDTO and TKA using the EOS imaging system. The secondary objective is to investigate the relationship between coronal and sagittal alignment parameters.

**Patients and methods:**

A total of 102 patients, including 51 who underwent OWDTO and 51 who underwent TKA, were included. Radiographic evaluations were performed using the EOS imaging system pre-operatively and at 12 months post-operatively. Coronal alignment parameters (medial proximal tibial angle (MPTA), coronal hip-knee-ankle angle (c-HKA), pelvic obliquity (PO), and sagittal alignment including sagittal HKA (s-HKA), pelvic incidence (PI), pelvic tilt (PT) and sacral slope (SS)) were assessed. Changes (Δ) and their correlations were analyzed. To account for potential confounding by age, sex, and Kellgren-Lawrence grade, partial correlation analysis adjusting for these variables was additionally performed.

**Results:**

TKA resulted in significant improvements in coronal and sagittal alignment parameters except PT (p < 0.001). OWDTO showed significant changes in coronal parameters except PO (p < 0.001), with no significant changes observed in sagittal alignment. Δs-HKA was weakly and moderate negatively correlated with Δc-HKA, ΔSS and ΔPO (r = −0.20, −0.22 and −0.33, 95%CI: −0.38 to −0.01, −0.40 to −0.03 and −0.49 to −0.14, *p* = 0.04, 0.03 and 0.001). ΔSS was weakly negatively correlated with the change in ΔMPTA (r = −0.24, 95%CI: −0.44 to −0.05, *p* = 0.02). ΔPI was strongly positively correlated with the change in ΔPT (r = 0.77, 95%CI: 0.67 to 0.83, *p* < 0.001). After adjusting for age, sex, and K-L grade, the correlations between changes in coronal and sagittal alignment parameters remained largely consistent with the unadjusted results.

**Conclusion:**

TKA resulted in greater improvements in both coronal and sagittal alignment parameters compared to OWDTO. Among sagittal parameters, the change in s-HKA showed the strongest, although modest, correlation with coronal alignment changes. The EOS imaging system may serve as a useful tool for simultaneously and reproducibly assessing coronal and sagittal alignment in patients undergoing OWDTO and TKA.

## 1 Introduction

Total knee arthroplasty (TKA) and various type of tibial osteotomy, including open wedge high tibial osteotomy (OWHTO), open wedge distal tuberosity osteotomy (OWDTO) and closed wedge high tibial osteotomy, are performed in patients with medial knee osteoarthritis (KOA) patients and have been shown to lead to favorable clinical outcome.^1^^,^^2^ Regarding the difference between TKA and OWHTO, TKA is typically performed for end-stage KOA, whereas OWHTO is used for early-stage KOA. Both surgical procedures are effective in treating KOA and aim to correct deformity to achieve neutral alignment or Fujisawa point.^3^, ^4^, ^5^.

Patients with KOA experience not only coronal alignment but also sagittal alignment problem, particularly due to an associated spinal sagittal problem known as knee-spine syndrome.^6^ Lumbar spine compensation occurs as KOA progresses, leading to the development of this syndrome.^7^ However, the relationship between pelvic-lower limb in coronal and in sagittal alignment after OWDTO and TKA remain unclear. In particular, it is challenging to evaluate this alignment in standing position using conventional imaging method like computed tomography (CT).

The EOS imaging system (EOS Imaging, Paris, France) is a low-dose imaging tool designed to evaluate three-dimensional standing-weighted whole-body alignment.^8^^,^^9^ It can generate 3D models using the ster-EOS 3D workstation.^10^^,^^11^ This system captures antero-posterior (AP) views, lateral long leg radiographs (LLRs), and full-body radiographs while the patient is standing. One of the main advantages of the EOS imaging system over traditional X-rays and CT is its ability to expose patients to a lower dose of radiation while using collimators to generate parallel beams.^12^ However, there are no reports of the evaluation in pelvic-lower limb alignment compared to both OWDTO and TKA using the EOS imaging system.

The primary objective of this study is to evaluate the pelvic–lower limb alignment after OWDTO and TKA using the EOS imaging system. The secondary objective is to investigate the relationship between coronal and sagittal alignment parameters. The hypothesis is that both OWDTO and TKA would improve the pelvic-lower limb alignment. Furthermore, the change in the medial proximal tibial angle (MPTA) would influence the sagittal alignment more than other coronal parameters.

## 2 Materials and methods

### 2.1 Patient selection

This retrospective study, based on prospectively collected data, was approved by our institution ethics committee (IRB No. E22-0166.). All procedures were performed in accordance with the ethical standards of the institutional research committee and with the 1964 Declaration of Helsinki. A total of 102 patients who underwent either TKA or OWDTO at our institution between January 2022 and August 2023 were included. Each patient contributed only one operated knee to the analysis; therefore, no adjustment for bilateral limb correlation was necessary. All patients had available follow-up data both pre-operative and 12 months post-operatively. The inclusion criteria were as follows: patients who underwent primarily unilateral TKA or OWDTO for medial KOA. The exclusion criteria were as follows: Other types of osteotomies, TKA for lateral KOA, revision TKA or OWDTO, TKA following prior tibial osteotomy, patients lost to follow-up, and those with a history of spinal surgery.

### 2.2 Radiological evaluation

Radiographic evaluations were conducted using the EOS imaging system, and 3D models were reconstructed using ster-EOS 3D workstation. Radiographic measurements were carried out by two surgeons (YK and KN). Key radiological parameters were assessed coronal alignment including MPTA, coronal hip-knee-ankle angle (c-HKA), pelvic obliquity (PO), and sagittal alignment including sagittal HKA (s-HKA), pelvic incidence (PI), pelvic tilt (PT) and sacral slope (SS). The intra- and inter-observer reliabilities were excellent, with ICC values exceeding 0.80 for all parameters.

### 2.3 Surgical procedures

The surgical procedure of OWDTO followed established protocols.^13^ All OWDTO procedures were performed using locking plate ((TriS medial HTO plate® (Olympus Terumo Biomaterials, Tokyo, US)). Patients were allowed to start with touch weight bearing, then to engage in partial weight bearing with crutches after one week, and finally to advance to full weight-bearing after two weeks. Additionally, they were instructed to begin range of motion (ROM) exercises within 90° after two weeks and were cleared for full ROM after four weeks. The surgical procedure of TKA followed established protocols.^14^ All TKA surgeries were performed using posterior stabilized TKA ((Vanguard PSRP Knee® (Zimmer Biomet, Indiana, USA)). The patients were allowed full weight bearing immediately.

### 2.4 Statistical analysis

Statistical analyses were performed using the GraphPad Prism Biostatistics software program (GraphPad Software Inc., La Jolla, CA, USA). A priori power analysis was performed using G*Power (version 3.1.9.6; Franz Paul, Kiel, Germany) to determine the required sample size. Based on effect size of 0.5, an alpha level of 0.05, and power of 0.80, a minimum of 51 patients per group was required to achieve sufficient statistical power. To meet the assumptions of the statistical analyses employed, we equalized the sample sizes across conditions. Demographic findings, radiographic parameters are presented with descriptive statistics as the mean and standard deviation. The Mann–Whitney *U* test and chi-square test were used to assess differences between OWDTO group and TKA group. The correlation between the change of the coronal and sagittal parameters was assessed by Pearson's correlation coefficient. To account for potential confounding by age, sex, and Kellgren-Lawrence grade, partial correlation analysis adjusting for these variables was additionally performed. Changes were defined as the difference (Δ) between the value pre-operatively and that 12 months post-operatively. Statistical significance was assumed at p values of < 0.05.

## 3 Results

### 3.1 Patients’ demographics

A total of 102 patients were included in this study. Among them, 51 underwent OWDTO with a mean age of 56.5 ± 8.0 years, and 51 underwent TKA with an mean age of 72.7 ± 8.2 years. The gender distribution for OWDTO patients was 24 females (47.1%) and 27 males (52.9%), and for TKA patients, it was 10 males (19.6%) and 41 females (80.4%). The mean body mass index (BMI) was 26.0 ± 4.4 kg/m^2^ in the OWDTO groups and 26.9 ± 5.1 kg/m^2^ in the TKA patients. The patient demographics is summarized in Table 1.

**Table 1:** Demographic characteristics of patients.

	DTO (n = 51)	TKA (n = 51)	*p*-value
Age, y	56.5 ± 8.0	72.7 ± 8.2	<0.0001*
Sex, n	Male 24/Female 27	Male 10/Female 41	<0.0001*
Height, cm	164.7 ± 8.8	156.7 ± 9.8	<0.0001*
Body weight, kg	70.1 ± 13.4	65.8 ± 11.6	0.09
BMI, kg/m^2^	26.0 ± 4.4	26.9 ± 5.1	0.30
K-L grade R/L	grade 1- 0 grade 2- 27 grade 3- 21 grade 4- 3	grade 1- 0 grade 2- 0 grade 3- 13 grade 4- 38	<0.0001*

※BMI: Body Mass Index, K-L: Kellgren-Lawrence, BMI: Body mass index.

### 3.2 Post-operative coronal alignment parameters assessed by EOS imaging system

**Fig. 1:**
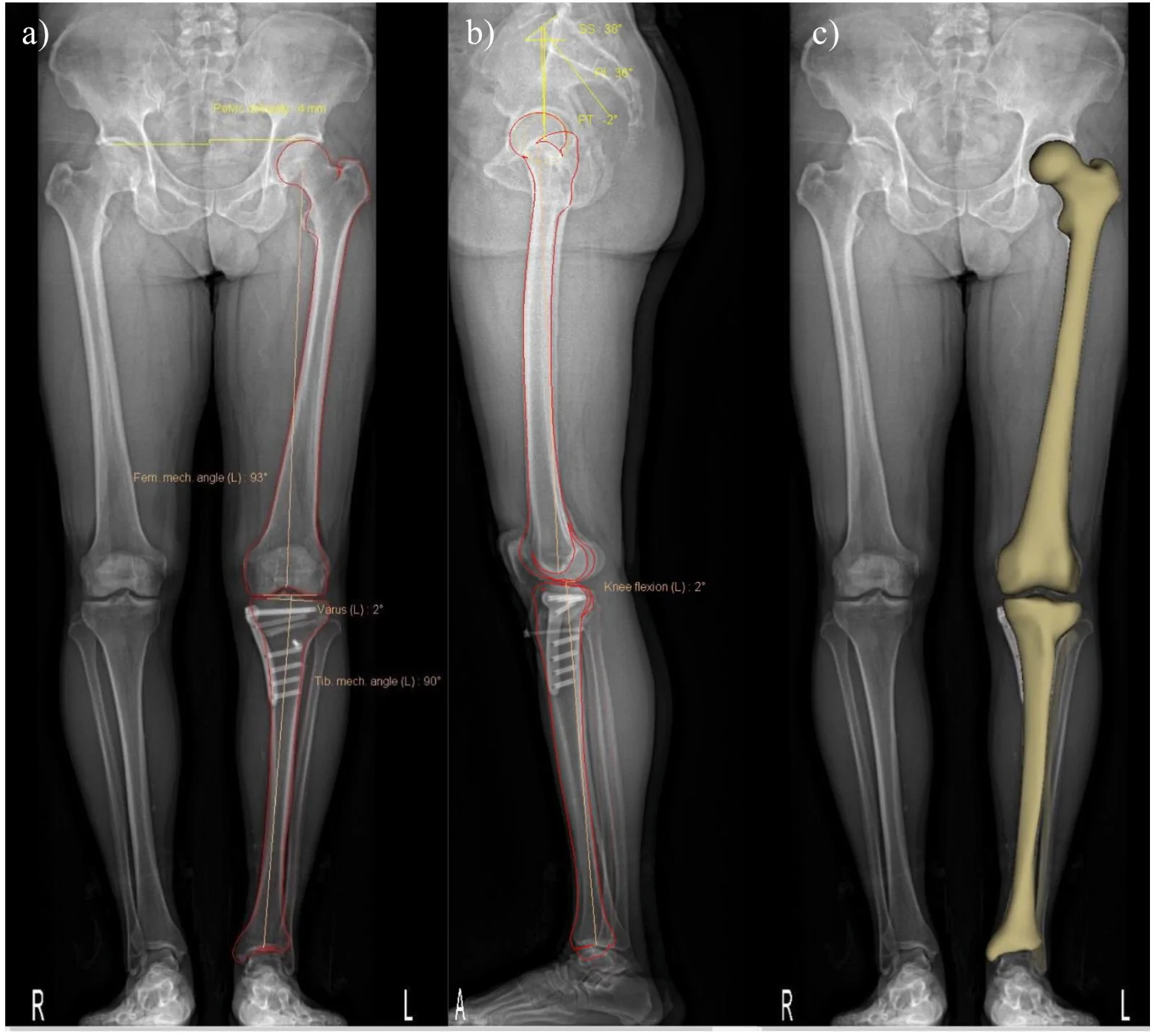
Radiographic measurements of lower limb alignment and spinopelvic parameters after open wedge distal tibial osteotomy using the EOS system (a) Coronal alignment was assessed based on spinopelvic parameters, including pelvic obliquity, and lower limb alignment. Knee varus and valgus were defined using the coronal hip–knee–ankle angle (cHKA), while additional alignment measures included the mechanical lateral distal femoral angle (mLDFA) and the medial proximal tibial angle (MPTA). (b) Sagittal alignment was assessed based on spinopelvic parameters– pelvic incidence (PI), sacral slope (SS), pelvic tilt (PT)-as well as lower limb alignment including the sagittal hip-knee-angle (s-HKA). (c) 3D reconstructed models using the sterEOS workstation.

In the OWDTO group, significant improvements in coronal alignment parameters, except for the mLDFA and PO. The MPTA increased from 83.7 ± 2.4° to 88.8 ± 8.2° (*p* < 0.0001), and the c-HKA increased from −5.1 ± 3.2° to 1.4 ± 2.3° (*p* < 0.0001). In the TKA group, all coronal parameters improved significantly. MPTA increased from 82.7 ± 3.8° to 87.5 ± 8.4° (p < 0.0001), mLDFA increased from 88.7 ± 1.9° to 90.0 ± 1.8° (*p* = 0.04), c-HKA increased from −8.2 ± 14.5° to −2.3 ± 3.5° (*p* < 0.0001), and PO increased from −0.6 ± 2.4 mm to 0.8 ± 2.3 mm (*p* < 0.0001) (Table 2). Fig. 1 shows radiographic measurements obtained using EOS imaging system and presents 3D reconstruction generated with the ster-EOS 3D workstation.

**Table 2:** Coronal alignment assessed using the EOS imaging system.

		Pre-operative	Post-operative 12 months	*p*-value
OWDTO	MPTA (°)	83.7 ± 2.4	88.8 ± 8.2	<0.0001*
	mLDFA (°)	88.9 ± 2.4	88.9 ± 2.4	-
	Coronal HKA (°)	−5.1 ± 3.2	1.4 ± 2.3	<0.0001*
	PO (mm)	0.3 ± 1.8	0.5 ± 1.7	0.51
TKA	MPTA (°)	82.7 ± 3.8	87.5 ± 8.4	<0.0001*
	mLDFA (°)	88.7 ± 1.9	90.0 ± 1.8	0.04*
	Coronal HKA (°)	−8.2 ± 14.5	−2.3 ± 3.5	<0.0001*
	PO (mm)	−0.6 ± 2.4	0.8 ± 2.3	<0.0001*

※OWDTO: Open wedge distal tuberosity osteotomy, TKA: Total knee arthroplasty, MPTA: Medial proximal tibial angle, %MA: % Mechanical axis, HKA: Hip knee angle, PO: Pelvic obliquity.

### 3.3 Post-operative sagittal alignment parameters assessed by EOS imaging system

In the OWDTO group, no significant changes were observed in any sagittal alignments. In contrast, the TKA groups showed significant improvements in sagittal alignments except for PT. The s-HKA decreased from 13.5 ± 6.9° to 8.3 ± 5.6° (*p* < 0.001), the PI increased from 59.7 ± 10.0° to 62.0 ± 10.6° (*p* = 0.04), and SS increased from 33.3 ± 9.6° to 36.4 ± 9.4° (*p* < 0.0001). No significant changes were observed in the PT (Table 3).

**Table 3:** Sagittal alignment assessed using the EOS imaging system.

		Pre-operative	Post-operative 12 months	*p*-value
OWDTO	s-HKA (°)	6.5 ± 5.5	6.8 ± 7.2	0.69
	PI (°)	56.1 ± 10.5	55.5 ± 9.6	0.47
	PT (°)	19.8 ± 12.6	18.0 ± 8.4	0.22
	SS (°)	34.2 ± 10.2	34.4 ± 10.1	0.89
TKA	s-HKA (°)	13.5 ± 6.9	8.3 ± 5.6	<0.0001*
	PI (°)	59.7 ± 10.0	62.0 ± 10.6	0.04*
	PT (°)	21.0 ± 9.3	22.0 ± 11.7	0.54
	SS (°)	33.3 ± 9.6	36.4 ± 9.4	0.04*

※OWDTO: Open wedge distal tuberosity osteotomy, TKA: Total knee arthroplasty, s-HKA: sagittal hip knee angle, PI: Pelvic incidence, PT: Pelvic tilt, SS: Sacral slope.

### 3.4 Correlation between coronal and sagittal alignment parameters

Δs-HKA was weakly and moderate negatively correlated with Δc-HKA, ΔSS and ΔPO (r = −0.20, −0.22 and −0.33, 95%CI: −0.38 to −0.01, −0.40 to −0.03 and −0.49 to −0.14, *p* = 0.04, 0.03 and 0.001). ΔSS was weakly negatively correlated with the change in ΔMPTA (r = −0.24, 95%CI: −0.44 to −0.05, *p* = 0.02). ΔPI was strongly positively correlated with the change in PT (ΔPT) (r = 0.77, 95%CI: 0.67 to 0.83, *p* < 0.001). After adjusting for age, sex, and K-L grade, the correlations between changes in coronal and sagittal alignment parameters remained largely consistent with the unadjusted results (Table 4 and supplemental table).

**Table 4:** The correlation between changes in sagittal and coronal alignment parameters.

	Unadjusted			Adjusted		
	r	95%CI	p value	r	95%CI	p value
ΔMPTA						
Δs-HKA	−0.16	−0.34 to 0.04	0.12	−0.15	−0.34 to 0.05	0.14
ΔPI	−0.16	−0.35 to 0.04	0.10	−0.16	−0.34 to 0.04	0.12
ΔPT	−0.13	−0.32 to 0.07	0.19	−0.09	−0.28 to 0.11	0.40
ΔSS	−0.24	−0.44 to −0.05	0.02*	−0.27	−0.44 to −0.07	0.01*
ΔmLDFA						
Δs-HKA	−0.29	−0.54 to 0.02	0.07	−0.19	−0.11 to 0.28	0.37
ΔPI	0.19	−0.12 to 0.47	0.22	0.15	−0.05 to 0.34	0.15
ΔPT	−0.05	−0.36 to 0.25	0.74	−0.11	−0.09 to 0.31	0.26
ΔSS	0.23	−0.08 to 0.51	0.14	0.16	−0.14 to 0.26	0.55
Δc-HKA						
Δs-HKA	−0.20	−0.38 to −0.01	0.04*	−0.21	−0.39 to −0.01	0.04*
ΔPI	0.01	−0.19 to 0.20	0.96	0.01	−0.18 to 0.21	0.90
ΔPT	−0.01	−0.20 to 0.19	0.96	0.00	−0.19 to 0.20	0.96
ΔSS	0.02	−0.18 to 0.21	0.85	−0.00	−0.20 to 0.19	0.97
ΔPO						
Δs-HKA	−0.33	−0.49 to −0.14	0.001*	−0.26	−0.43 to −0.06	0.01*
ΔPI	−0.11	−0.30 to 0.09	0.28	−0.15	−0.33 to 0.05	0.15
ΔPT	−0.04	−0.23 to 0.15	0.68	−0.06	−0.25 to 0.14	0.58
ΔSS	0.17	−0.02 to 0.36	0.08	0.17	−0.03 to 0.36	0.09

※*Adjusted for age, sex, and Kellgren-Lawrence grade using partial correlation analysis.

MPTA: Medial proximal tibial angle,s, HKA: Hip knee angle, PO: Pelvic obliquity, s- PI: Pelvic incidence, PT: Pelvic tilt, SS: Sacral slope.

## 4 Discussion

The main finding of this study demonstrated that both coronal and sagittal alignment parameter-except for PT-significantly improved 12 months after TKA. In contrast, coronal alignment parameters, except for PO, were significantly changed 12 months after OWDTO, while sagittal alignment parameters remained unchanged. Among sagittal parameters, changes in s-HKA showed the strongest correlation with coronal alignment.

Contrary to our hypothesis, our results showed that the change in s-HKA, rather than coronal parameters such as MPTA or c-HKA, demonstrated the strongest correlation with sagittal alignment changes. This finding suggests that correction of sagittal knee flexion contracture may be more critical than correction of coronal malalignment in driving pelvic-spinal compensation, consistent with the concept of knee-hip-spine syndrome.^6^ This highlights the clinical importance of addressing flexion contracture, rather than coronal deformity alone, in surgical planning for both OWDTO and TKA.

Although EOS imaging system is expensive and not widely available, it offers valuable advantages as a low-dose imaging modality in clinical orthopedics.^10^^,^^15^ The system captures two perpendicular X-ray beams simultaneously, allowing full-body 3D reconstructions in the standing position, without magnification. Compared to conventional radiography and CT, the EOS imaging system provide several benefits, including low dose radiation, shorter exposure time, full body image in full weight bearing, 3D model reconstruction, and automated alignment parameter analysis.^12^^,^^16^^,^^17^ It has proven utility in the evaluation of scoliosis, torsional deformity, and total hip arthroplasty.^18^^,^^19^ More recently, it has also been applied to research in KOA.^20^

The previous study suggested the patients undergoing total joint arthroplasty often have a high prevalence of adult spinal deformity.^21^ Among the pelvic parameters, an abnormal PI has been associated with suboptimal outcomes in total hip arthroplasty and primary TKA.^21^^,^^22^ Therefore, pre-operative assessment and surgical planning should consider not only lower alignment but also pelvic alignment.

Both OWDTO and TKA mainly aim to correct coronal malalignment can also change following correction of coronal deformities.^23^^,^^24^ Sagittal lower limb alignment changes follow correcting the coronal lower-limb alignment. According to Yoo et al., flexion in sagittal alignment was significantly changed post 1 year after TKA.^25^ Similarly, Kim et al. also reported that s-HKA angle in sagittal alignment was changed.^26^ This study results are consistent with prior studies which reported changes in sagittal flexion and s-HKA post-operatively.

Regarding pelvic sagittal alignment, according to Lee et al.,^27^ SS increased significantly after TKA in patients with KOA and strong contracture flexed knee. Kim et al.^26^ reported that correction varus alignment following TKA led to significant decreased PT and increased SS in the sagittal alignment. In this study, both SS and PI significantly changed after 12 months after TKA. However, the magnitude of PI change was small (approximately 2.3°), and although statistically significant, this change may not necessarily reflect a clinically or anatomically meaningful alteration, given that PI is generally considered a relatively stable parameter after skeletal maturity. Although PI was once believed to be a fixed anatomical parameter after skeletal maturity, recent study suggest it may change via functional movement at the sacroiliac joint.^28^ Several studies have reported PI changes after long fusions to the pelvis.^29^^,^^30^ This study revealed that TKA leads to change pelvic alignment over 12 months. In this context, we hypothesize that correction of lower limb alignment via TKA may indirectly influence pelvic alignment through ligamentous tension and biomechanical adaptation.

Although the change in sagittal pelvic-lower limb alignment by TKA is well documented, the relationship between parameters in pelvic-lower limb coronal and in sagittal alignment in OWDTO remains unclear. OWDTO did not significantly change sagittal alignment parameters in this study. This discrepancy likely reflects two main factors. First, OWDTO involves correction limited to the tibia, whereas TKA involves combined femoral and tibial correction, potentially producing a greater overall effect on pelvic sagittal alignment. Second, patients undergoing OWDTO were younger and had less severe osteoarthritis, and were therefore less likely to have preoperative knee flexion contracture—a compensatory response to age-related spinal-pelvic deformity^31^^,^^32^ —or the associated pelvic retroversion and increased pelvic tilt. In contrast, older patients with more advanced osteoarthritis undergoing TKA more frequently presented with such contractures and compensatory pelvic changes preoperatively, providing greater potential for postoperative sagittal realignment.

This study has several limitations. First, the follow-up duration was relatively short. Second, we focused solely on pelvic and lower limb alignment rather than on whole-body sagittal alignment. Third, clinical outcomes were not analyzed in relation to radiographic findings. Fourth, the OWDTO and TKA groups differed substantially in age, sex distribution, and K-L grade, which may have confounded the observed associations between coronal and sagittal alignment changes. To address this, we performed partial correlation analyses adjusting for these variables (Table 4); the correlations of primary interest remained largely consistent, suggesting they were not solely attributable to baseline differences. Nevertheless, residual confounding cannot be fully excluded given the retrospective, non-matched design, which may also have introduced selection bias; future studies using matched cohorts would help clarify the independent contribution of surgical procedure type. Potential measurement errors inherent to EOS imaging could also have affected the accuracy of alignment assessments. Although several correlations reached statistical significance, most were weak to moderate in magnitude, and their clinical or biomechanical significance should be interpreted with caution given the relatively large sample size, which increases sensitivity to detect small effect sizes.

## 5 Conclusion

TKA resulted in greater improvements in both coronal and sagittal alignment parameters compared to OWDTO. Among sagittal parameters, the change in s-HKA showed the strongest, although modest, correlation with coronal alignment changes. The EOS imaging system may serve as a useful tool for simultaneously and reproducibly assessing coronal and sagittal alignment in patients undergoing OWDTO and TKA.

## Authors’ contributions

YK wrote the manuscript text and prepared the tables. JT, KN, KY, SH, JS, HK, YS and MK, contributed substantially to the manuscript's drafting. MI supervised the research. All authors reviewed and approved the final manuscript.

## Level of evidence

Level Ⅲ, Retrospective case–control study.

## Funding

The correct funding source name is the Japan Society for the Promotion of Science (JSPS), and the grant type is Grant-in-Aid for Young Scientists (grant no.23K15725).

## Conflict of interest

The author(s) have no conflicts of interest relevant to this article.
