# Supplementary material for: Comparison of coronal and sagittal pelvic-lower limb alignment after open wedge distal tibial tuberosity osteotomy and total knee arthroplasty using the EOS imaging system
Source: Asia Pac J Sports Med Arthrosc Rehabil Technol. 2026 Aug 19;46:1–6. doi: 10.1016/j.asmart.2026.08.002 (PMC13519259; doi:10.1016/j.asmart.2026.08.002)
Supplement: Supplementary file 1 [file mmc1.docx]

Supplementary Table. The correlation between changes in sagittal alignment parameters

|  | Unadjusted | | | Adjusted | | |
| --- | --- | --- | --- | --- | --- | --- |
|  | r | 95%CI | p value | r | 95%CI | p value |
| Δs-HKA |  |  |  |  |  |  |
| ΔPI | -0.05 | -0.24 to 0.15 | 0.61 | -0.01 | -0.21 to 0.19 | 0.91 |
| ΔPT | -0.10 | -0.29 to 0.10 | 0.33 | -0.09 | -0.28 to 0.11 | 0.40 |
| ΔSS | -0.22 | -0.40 to -0.03 | 0.03* | -0.20 | -0.38 to -0.00 | 0.045* |
| ΔPI |  |  |  |  |  |  |
| ΔPT | 0.77 | 0.67 to 0.83 | 0.001* | 0.75 | 0.65 to 0.85 | 0.001* |
| ΔSS | 0.04 | -0.16 to 0.23 | 0.69 | 0.04 | -0.16 to 0.24 | 0.70 |
| ΔPT |  |  |  |  |  |  |
| ΔSS | -0.10 | -0.29 to 0.09 | 0.31 | -0.10 | -0.30 to 0.09 | 0.30 |

*※*Adjusted for age, sex, and Kellgren-Lawrence grade using partial correlation analysis.*

*PO: Pelvic obliquity, s-HKA: sagittal hip knee angle, PI: Pelvic incidence, PT: Pelvic tilt, SS: Sacral slope*
